# Investigation of Hydrogen Gas Sensing Properties of WSe_2_, Ag_2_Se, WSe_2_/Ag_2_Se Nanostructured Electrodes Synthesized by Electrochemical Methods

**DOI:** 10.1021/acsomega.5c11524

**Published:** 2026-01-06

**Authors:** Fatma Bayrakçeken Ni̇şancı

**Affiliations:** Faculty of Science, Department of Chemistry, 37503 Ataturk University, 25240 Erzurum, Turkey

## Abstract

In this study, WSe_2_, Ag_2_Se, and WSe_2_/Ag_2_Se nanostructured electrodes were synthesized via an electrochemical deposition method, and their hydrogen gas sensing performances were systematically investigated. The synergistic interaction between WSe_2_ nanosheets and Ag_2_Se nanoparticles significantly enhanced charge transport and catalytic activity in the hybrid electrode. Compared to the pristine WSe_2_ and Ag_2_Se electrodes, the hybrid electrode exhibited a remarkably higher current response of 7.89 mA cm^–2^ at 5000 ppm of H_2_ and demonstrated stable and reversible sensing behavior over a wide range of hydrogen concentrations (1000–5000 ppm). Analyses of SEM, Raman, and UV–Vis data revealed an increased surface area and improved electronic coupling within the hybrid structure. Furthermore, EIS and OCP measurements confirmed accelerated interfacial charge transfer and superior electrochemical stability. Overall, this study introduces a novel electrochemically synthesized WSe_2_/Ag_2_Se hybrid nanostructure exhibiting excellent sensing performance at low temperatures (25–150 °C) and provides a promising strategy for the development of low-power, highly stable hydrogen gas sensors.

## Graphical abstract

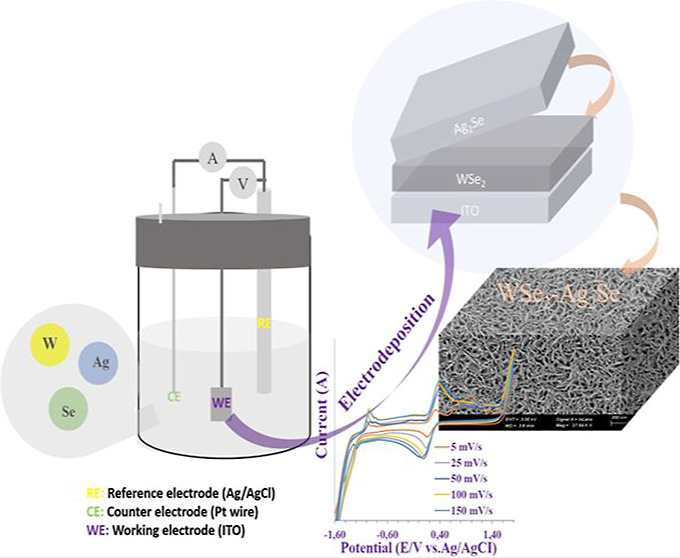

## 1 Introduction

Nanostructured materials with excellent properties, such as Ag_2_Se and WSe_2_, are metal selenides with large magnetoresistance and high electrical conductivity. Due to these properties, they are used for various applications such as gas sensors, electrochemical sensors, dopamine detectors, and anodes for Li-ion batteries.^1^ Hydrogen, which is widely used as a renewable fuel, is becoming increasingly important because it does not pollute the air and is easy to create.^2,3^ New technologies and devices that can work with this fuel are being developed. Especially in recent years, the materials used as hydrogen sensors have been electrochemical, catalytic, thermal conductivity, surface acoustic wave, metal oxide, and resistive metal-based hydrogen sensors according to the gas sensing mechanism^4–13^ Designing and manufacturing hydrogen sensors capable of detecting low-concentration hydrogen gas leakage is also important.^14,15^ In particular, electrochemical hydrogen sensors are widely used commercially in industry. Electrochemical sensors^16,21^ require very little power to operate, the response time is fast, and the life expectancy of the sensor is highly dependent on environmental pollutants, temperature, and humidity.

In recent years, two-dimensional transition metal dichalcogenides (TMDs) such as MoS_2_, WS_2_, and WSe_2_ have attracted significant attention as promising materials for gas sensing applications owing to their high surface-to-volume ratio, tunable band gaps, and abundant active sites, which facilitate efficient charge-transfer interactions with gas molecules.^22–24^ Their unique layered structures allow strong adsorption of gas species and efficient modulation of electrical conductivity upon exposure, enabling sensitive detection even at low operating temperatures. Furthermore, recent studies have demonstrated that defect engineering, heterostructure formation, and hybridization of TMDs with noble metals or metal oxides can further enhance sensing performance by improving charge carrier mobility, catalytic activity, and surface reactivity.^25–27^ For example, MoS_2_–Pd and WS_2_–ZnO composites have shown significantly enhanced response and selectivity toward hydrogen and volatile organic compounds under mild conditions.^25,28^

In this context, by synthesizing thin films based on electrochemical methods (WSe_2_/Ag_2_Se nanostructured electrodes on ITO Indium tin oxide substrates), we have developed performance sensing results in a wide temperature range and detailed behavior of previously unexplained stability and renewability have been fully clarified. The synergistic interfacial coupling between these two components is expected to promote rapid electron transfer and efficient hydrogen adsorption, leading to superior low-temperature gas sensing performance. In this sense, the electrochemical properties of the WSe_2_/Ag_2_Se nanostructured electrodes synthesized by electrochemical methods were improved by preparing electrodes with an increased active surface area using high-quality nanostructured materials. This approach not only enhances the optical stability of the material but also contributes to the advancement of hydrogen storage technology, which is recognized as a promising energy solution for the future, by addressing its existing limitations. Furthermore, the proposed electrochemical synthesis route offers a cost-effective and straightforward strategy for fabricating high-performance WSe_2_/Ag_2_Se electrodes directly at room temperature and ambient pressure, without the need for any additional thermal or chemical treatments. In addition, WSe_2_/Ag_2_Se nanostructures with desired composition, structure, and size were easily synthesized using this method by controlling electrochemical parameters such as potential and time. In addition to higher and more stable structural properties, the morphological properties of the nanostructured layers of the electrodes significantly affect the electrode performance. Since this thin film formation was carried out electrochemically directly on ITO, interfacial defects and other contact problems were minimized.^17^ In this method, the nanostructure’s shape, morphology, and dimensions to be synthesized can be easily controlled by changing electrochemical parameters such as deposition potential and time,^18–20^ current density, and electrolyte components. In addition, literature studies show that materials to be synthesized with different production methods can lead to different sensitivities. In particular, the electrochemical synthesis method can produce suitable surfaces with high precision at the atomic scale and precise film thickness control. The electrochemical deposition method has enabled the production of high-quality thin films at low temperatures due to the ability of precursors in the gas phase to adhere to the surface without reaching very high temperatures.^29–33^

Since most gas sensors have high operating temperatures, high power consumption, and high cost, the work to be done in this study becomes more important. Therefore, the design and fabrication of hydrogen sensors consisting of materials capable of sensing low hydrogen gas concentrations, especially at room temperature, has been realized. The schematic representation given in Figure 1 shows the H_2_ sensitivity of WSe_2_–Ag_2_Se thin film electrodes in the room WSe_2_ (15 min electrodeposition time) – Ag_2_Se (5 min electrodeposition time) thin film electrodes were analyzed electrochemically.

**Figure 1:**
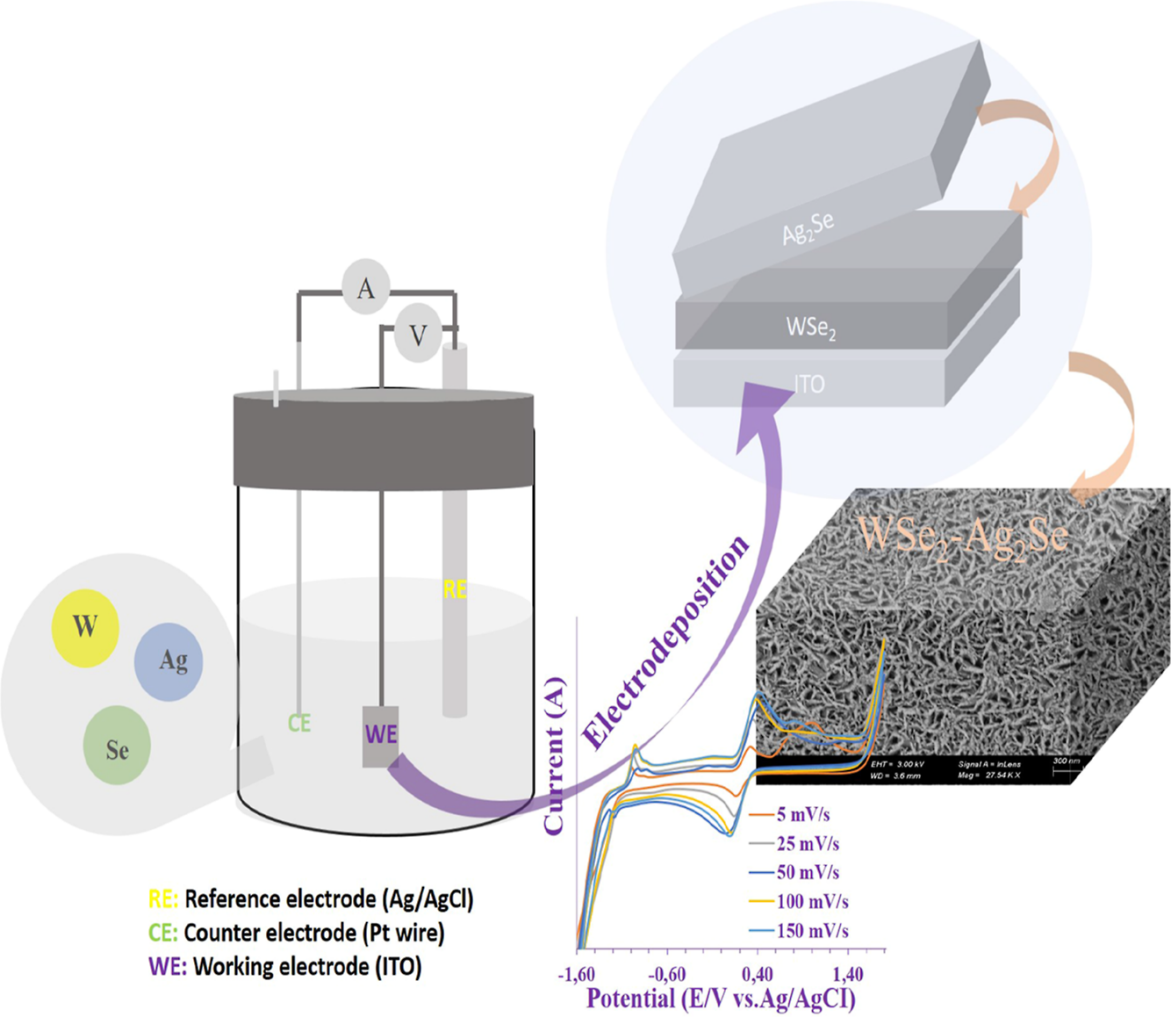
Electrochemical synthesis of ITO/WSe_2_/Ag_2_Se nanostructured arrays and schematic representation of sensitivity-time plot.

## 2 Experimental Section

A BAS 100B/W electrochemical workstation engaged to a three-electrode cell (C3 Cell Stand, BAS) was used for the electrochemical procedures. The powder X-ray diffractograms of the deposited nanostructures were enrolled using a Rigaku powder X-ray diffractometer with a Cu K-X-ray source (λ = 1.5406̊A). The morphological analysis and elemental composition determination WSe_2_, Ag_2_Se and WSe_2_–Ag_2_Se nanostructures were carried out by a ZEISS system coupled to the scanning electron microscope. Electrochemical measurements were implemented in a three-electrode electrochemical cell configuration using the WSe_2_, Ag_2_Se and WSe_2_–Ag_2_Se nanostructures deposited on ITO as a working electrode. This ability continues for WSe_2_ thin film electrodes; in a solution containing 0.5 mM Na_2_WO_4_·2H_2_O, 1 mM SeCl_4_ pH:5 acetate buffer at a −400 mV constant potential at 25 °C for 60 min electrodeposition time. Ag_2_Se thin film electrodes; in 0.211 g AgCl, 1 mM SeCl_4_, 0.011 g Na_2_SO_4_, 2.52 g KNO_3_, the coating process was carried out at −200 mV for 60 min electrodeposition time.

## 3 Results and Discussion

Figure 2 shows the SEM images of the (a) WSe_2_, (b) Ag_2_Se, and (c) WSe_2_/Ag_2_Se films prepared by electrochemical deposition. The pure WSe_2_ film exhibits a uniform and compact surface composed of circular nanosheets with an average lateral size of 50–80 nm. The Ag_2_Se film consists of densely packed spherical and flower-like nanoparticles with an average diameter of 100–150 nm. In the WSe_2_/Ag_2_Se composite, a mixed and porous morphology is observed, combining nanosheets and rod-like subunits with an estimated size of 120–200 nm.

**Figure 2:**
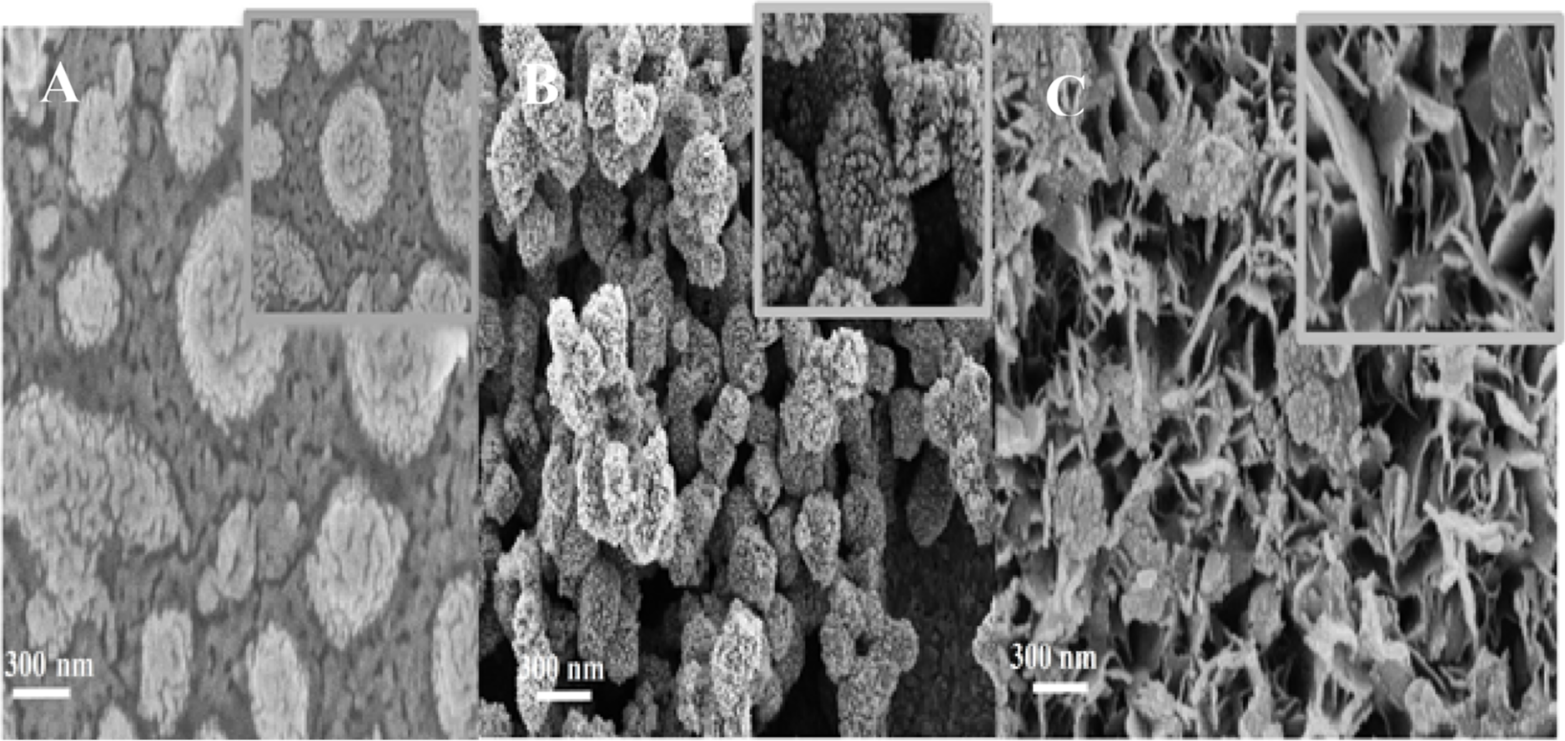
SEM images of (A) WSe_2_, (B) Ag_2_Se and (C) WSe_2_–Ag_2_Se nanostructures on ITO electrodes.

The transition from the smooth WSe_2_ morphology to the highly porous WSe_2_/Ag_2_Se hybrid structure confirms the successful formation of a synergistic heterojunction network that enhances gas-sensing performance. The hybrid architecture formed by WSe_2_ and Ag_2_Se increases the specific surface area and pore volume of the composite, providing a greater number of active sites and facilitating charge carrier transport during electrochemical reactions. Moreover, the strong interfacial interactions between the two phases promote the rapid adsorption and desorption of catalytic intermediates, thereby improving charge-transfer efficiency. Consequently, the robust electronic coupling established at the WSe_2_–Ag_2_Se interfaces plays a pivotal role in the remarkable enhancement of the hybrid nanostructure’s catalytic and gas-sensing performance.^34–36^

The XRD pattern of WSe_2_ was indexed to WSe_2_, JCPDS card No. 06-0080, which belongs to the hexagonal crystal system. The WSe_2_ thin film is homogeneous, free of cracks or pinholes, and adheres well to the ITO substrate, with spherical grains fused contiguously. The XRD pattern^34^ shows a diffraction peak at 2θ = 30.00°, corresponding to the WSe_2_ (100) plane, which exhibits the highest intensity peak. In addition, a diffraction peak corresponding to the (101) plane is observed at 2θ = 32.48°. Furthermore, Ag_2_Se matches well with orthorhombic Ag_2_Se (JCPDS No. 71-2410); for this material, the strong diffraction peak of the (102) plane at 2θ = 30.89° provides strong evidence for multilayer characteristics. The XRD patterns are predominantly indexed to Ag_2_Se,^37,38^ where weak reflections corresponding to the (111) plane at 2θ = 27.2° and very weak reflections corresponding to the (120) plane at 2θ = 31.40° can be identified (Figure 3). The XRD patterns of the thin and thick WSe_2_–Ag_2_Se hybrid films prepared at varying electrodeposition durations reveal diffraction peaks associated with both WSe_2_ and Ag_2_Se nanostructures, confirming the coexistence of the two crystalline phases. As a result, the hybrid materials are enriched with additional abundant reactive sites that can simultaneously allow fast charge transfer.^39,40^ Thus, the conductivity is significantly improved through electronic modulation, which is realized through a double-junction phase and interface engineering strategy. It can be well observed from the peaks of WSe_2_–Ag_2_Se that all diffraction peaks can be indexed as WSe_2_ or Ag_2_Se, indicating this process’s successful preparation of WSe_2_–Ag_2_Se.

**Figure 3:**
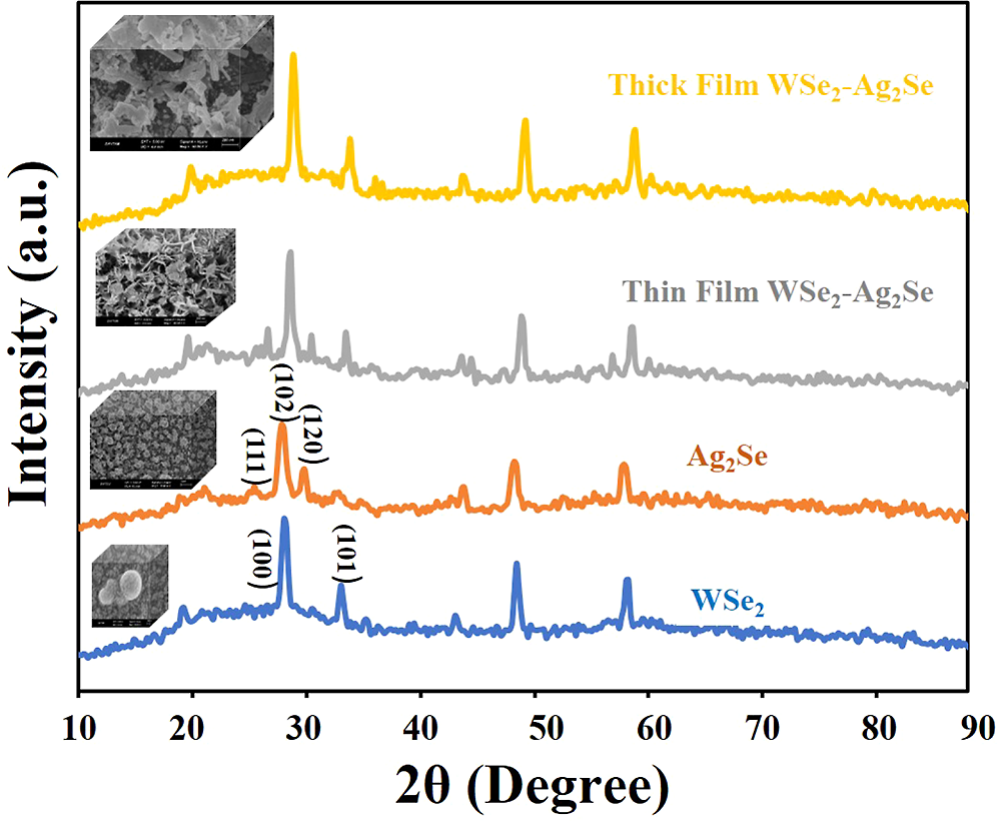
XRD data of WSe_2_, Ag_2_Se and WSe_2_–Ag_2_Se at different film thicknesses.

Figure 4 shows the optical absorption spectra of WSe_2_ nanosheets. As expected, three characteristic absorption peaks of WSe_2_ are observed in the range of 400–800 nm. The peaks at 567.92 and 594 nm correspond to direct excitonic transitions in WSe_2_, which arise from the energy splitting of the valence band caused by spin–orbit coupling. The absorption edge of WSe_2_ was observed around 567.92 nm, corresponding to an optical band gap of approximately 2.18 eV. Additionally, the peak observed at 417 nm is attributed to interband transitions occurring in few-layer WSe_2_ nanosheets.^41^ Furthermore, the formation of Ag_2_Se nanoparticles (NPs) exhibits a broad absorption band within the range of 367–595 nm, which can be attributed to nanoparticle agglomeration and the formation of larger grain sizes, corresponding to an optical band gap of approximately 2.08 eV.^40,41^

**Figure 4:**
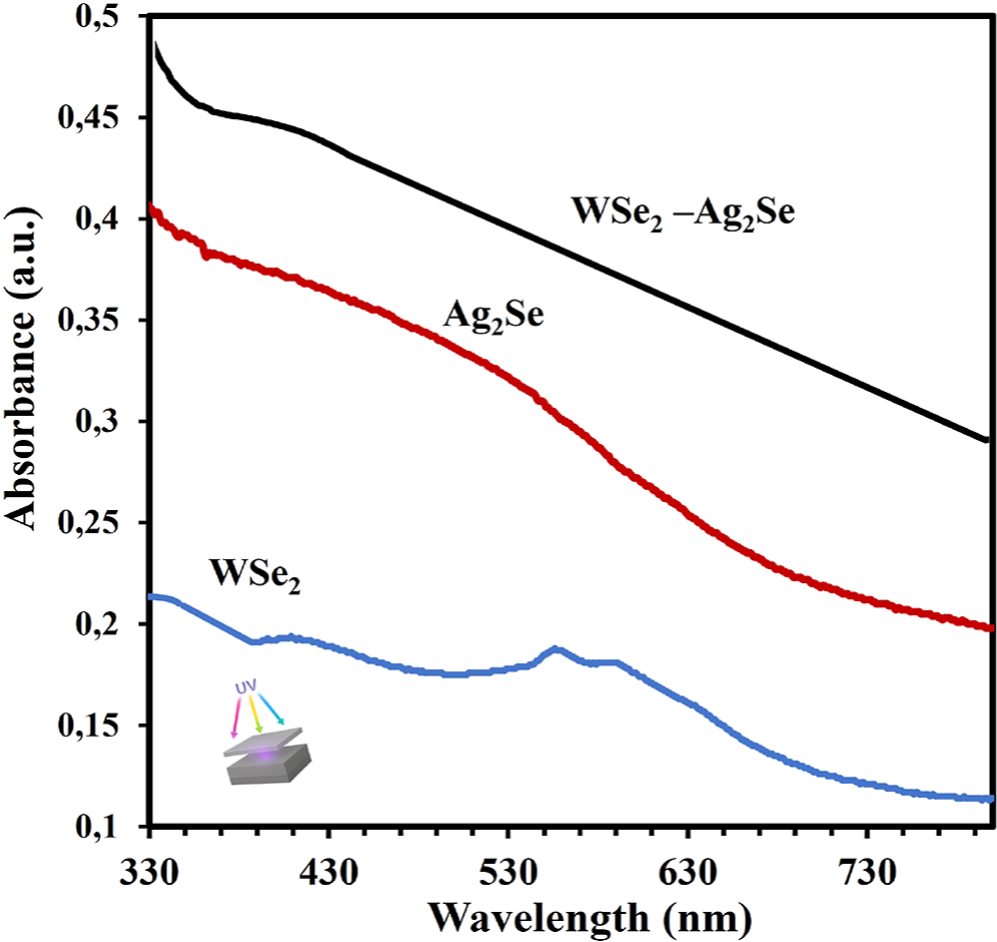
UV–visible absorption spectra of WSe_2_, Ag_2_Se and WSe_2_–Ag_2_Se nanostructures.

The formation of WSe_2_–Ag_2_Se nanostructures was confirmed by the appearance of an absorption band at approximately 413 nm within the range of 385–446 nm, consistent with previously reported literature values. Moreover, the unique structure of WSe_2_–Ag_2_Se provides a special material with better storage and catalytic performance. The WSe_2_–Ag_2_Se hybrid nanostructure exhibited a distinct absorption band centered at approximately 413 nm, showing a noticeable blue shift compared to pure WSe_2_. This shift indicates an increase in the optical band gap to about 3.00 eV, suggesting the presence of strong interfacial coupling and electronic interactions between WSe_2_ and Ag_2_Se. The observed spectral shift and enhanced absorption intensity confirm the successful formation of a new hybrid electronic state within the WSe_2_–Ag_2_Se system.

Partial XPS spectra for all the constituent elements are given for the hybrid nanostructures in Figure 5a,b. In Figure 5a, the chemical state of the electrochemically prepared Ag_2_Se thin film is analyzed by XPS. In the partial spectra of Ag_2_Se, the narrow scan of the Ag 3d spectrum shows two peaks at 367.74 and 373.72 eV, indicating the 3d5/2 and 3d3/2 levels, respectively (Figure 5a), indicating the Ag­(I) state. Figure 5a shows the high-resolution spectrum of Se 3d, showing two peaks at 53.6 and 54.4 eV corresponding to 3d5/2 and 3d3/2, indicating the −2 oxidation state of Se. Therefore, XPS confirms the existence of single-phase pure Ag_2_Se and is well supported by structural studies.^42,43^ Furthermore, the XPS part spectrum obtained from WSe_2_ in Figure 5b consists of two prominent peaks at 32.65 and 34.85 eV binding energies, which correspond well with W 4f_7/2_ and W 4f_5/2_ of the 2H phase.^36^

**Figure 5:**
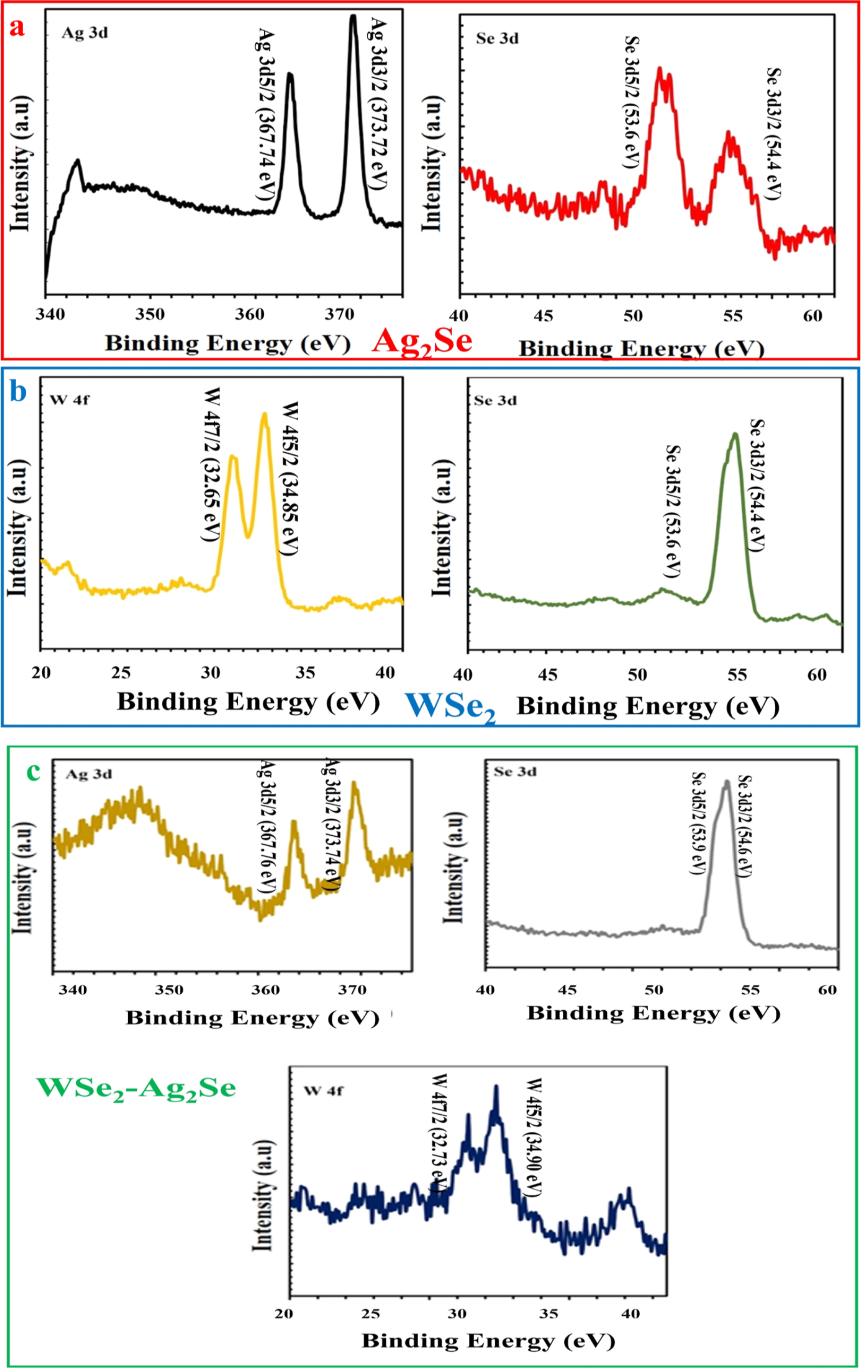
Partial scan XPS spectrum of (a) Ag 3d, Se 3d of Ag_2_Se, (b) W 4f, Se 3d of WSe_2_ and (c) Ag 3d, Se 3d and W 4f of WSe_2_–Ag_2_Se.

An XPS analysis was conducted to verify the presence of Ag/W/Se bonding in the WSe_2_–Ag_2_Se nanostructures, and the corresponding results are presented in Figure 5c. After the hybridization of WSe_2_ with Ag_2_Se, the characteristic W 4f_7/2_ and W 4f^5/2^ signals of WSe_2_ shifted to binding energies of 32.73 and 34.90 eV, respectively. The Se 3d spectrum exhibited doublet peaks at 53.90 and 54.60 eV, corresponding to the 3d_5/2_ and 3d_3/2_ states, which represent the binding energies of the hybrid nanosheet species. Ag_2_Se displayed two binding energy peaks at 367.74 and 373.72 eV, corresponding to Ag 3d_5/2_ and Ag 3d_3/2_, respectively. However, in the hybrid nanosheets, a slight shift in the Ag 3d binding energies (3d_5_/_2_ – 367.76 eV and 3d_3_/_2_ – 373.74 eV) was observed compared to those of Ag_2_Se nanocrystals. This shift indicates the occurrence of electronic interactions between Ag_2_Se and WSe_2_ at the interface/heterojunction within the hybrid nanosheets. Moreover, the selenide-related peaks in the hybrid nanosheets exhibited a shift in binding energies from their corresponding positions in Ag_2_Se and WSe_2_, gaining intermediate binding energies. It was also observed that the two doublets overlap more significantly, indicating a stronger contribution from the W–Se bond.

The hybridization of WSe_2_ with Ag_2_Se enhances its metallic nature and responds to the phase stabilization of WSe_2_ and Ag_2_Se nanosheets through interface interaction. Therefore, XRD, SEM, determinations, and XPS analysis consistently support the successful construction of heterostructured WSe_2_/Ag_2_Se hybrid nanosheets as van der Waals heterojunctions. Without sacrificing reagents or cocatalysts, the H_2_-current response was read for ten-second H_2_ gas on–off cycles at 25 °C at different temperatures and different flow rates at short-circuit potential in 0.10 M Na_2_SO_4_ electrolyte solution (Figure 6). Figure 6a shows that in the presence of H_2_ gas, the current response signals on WSe_2_, Ag_2_Se, and WSe_2_–Ag_2_Se thin film electrodes increased to 1.03 mA cm^–2^, 5.68 mA cm^–2^, 7.89 m cm^–2^, respectively. In the absence of H_2_ gas, the value of H_2_-current response signals decreases to zero. In Figure 6a, the best fast and smooth H_2_-current response signal value is seen in WSe_2_–Ag_2_Se thin film electrodes, and it is thought that the photocurrent responses of WSe_2_–Ag_2_Se thin film electrodes show the fast process of charge transfer and its relationship with the crystal structure and the increase of surface area.^39,44^

**Figure 6:**
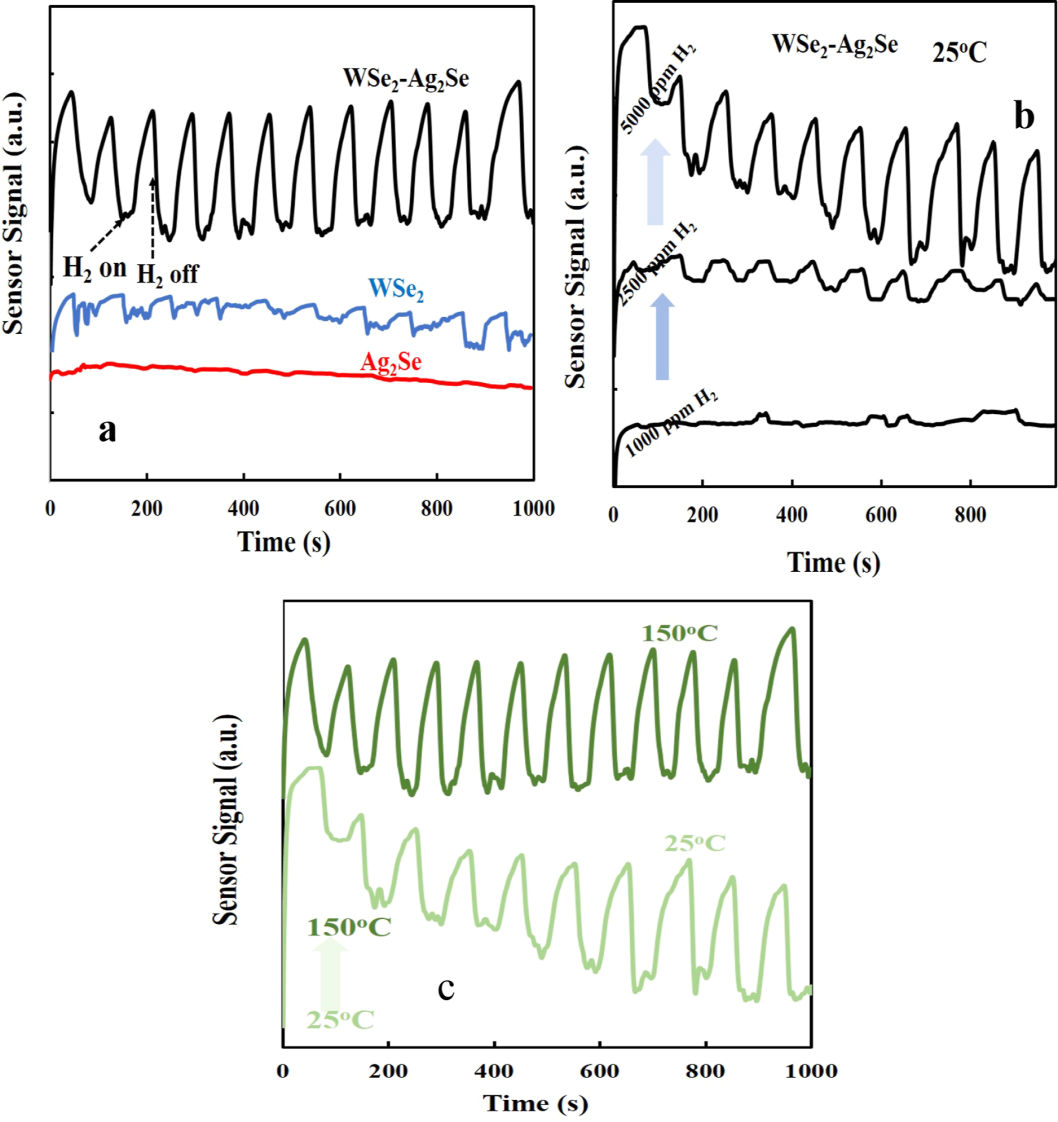
(a) Hydrogen response of Ag_2_Se, WSe_2_ and WSe_2_–Ag_2_Se thin films at 5000 ppm at 25 °C, (b) extreme response of WSe_2_–Ag_2_Se thin films at different H_2_ growth rates (hydrogen accumulations) from 1000 to 5000 ppm, and (c) signal change and hydrogen response of WSe_2_–Ag_2_Se thin films at 5000 ppm of H_2_ at operating temperatures such as 25 °C and 150 °C.

In addition, the current responses generated by WSe_2_–Ag_2_Se nanostructures are stable and reproducible over many cycles, indicating that the electrode is corrosion-free. Again, in Figure 6b, different H_2_ gas flow rates of 1000, 2500, and 5000 ppm were obtained on WSe_2_–Ag_2_Se thin film electrodes at 25 °C for ten-second H_2_ gas on–off cycles. Since the effect of WSe_2_–Ag_2_Se electrodes against gas is generally dependent on the interaction of the gas with the sensing film layer on the surface, the sensitivity value and response speed of WSe_2_–Ag_2_Se will improve with increasing surface area of the sensing film layer. The sensitivity increases with increasing H_2_ gas concentration in parallel with increasing flow rate (as gas concentration increases), and more hydrogen molecules interact with oxygen molecules attached to the surface. The results showed the best H_2_ current response values for WSe_2_–Ag_2_Se thin film electrodes at 5000 ppm compared to other flow rates. In addition, H_2_ current responses obtained on WSe_2_–Ag_2_Se thin film electrodes at different temperatures (from 25 to 150 °C) at 5000 ppm of H_2_ flow rates are shown in Figure 6c. When the current responses at 25 °C are compared with the H_2_ current response at 150 °C in Figure 6c, it is observed that the H_2_ current response increases with increasing temperature. However, the differences are not very high, indicating that WSe_2_–Ag_2_Se thin film electrodes can be easily sensitive to H_2_ gas at room temperature without the need to go to high temperatures. In H_2_ gas sensing, response and recovery times are key indicators of sensor efficiency and reliability. As shown in Figure 6, the WSe_2_/Ag_2_Se hybrid electrode demonstrates rapid and repeatable behavior with response and recovery times of approximately 35 and 40 s, respectively. This fast dynamic indicates efficient charge transfer and reversible surface reactions, ensuring stable and reproducible sensing performance.

In the literature, the operating temperature of most H_2_ sensors is relatively high, and the high operating temperature causes high power consumption and costs. However, the electrodes obtained in this study overcome these problems and enable the development of room temperature sensitive, low power consumption H_2_ gas sensors.^9,11,45^

Electrochemical impedance spectroscopy (EIS) and open-circuit potential (OCP) analyses also support this mechanism (Figure 7). Nyquist plots obtained for WSe_2_, Ag_2_Se, and WSe_2_–Ag_2_Se thin films prepared on ITO electrode in 0.1 M KCl solution containing 10 mM Fe­(CN)_6_^3–^/Fe­(CN)_6_^4–^ are shown in Figure 7a. The Nyquist plots are fitted according to the electrical circuit in Figure 7a. Here, the faradaic electron transfer resistance (*R*_p_) corresponds to the diameter of the semicircle formed. The solution resistance (*R*_w_) is the intersection point of the True *Z*′ axis of the graph. The constant phase element (CPE) is the capacitance of the double layer.^46,47^ The charge transfer resistance (*R*_p_) decreased notably from 317 Ω for WSe_2_ and 282 Ω for Ag_2_Se to 259 Ω for the WSe_2_/Ag_2_Se hybrid, confirming faster interfacial electron exchange due to the synergistic coupling between the two phases.

**Figure 7:**
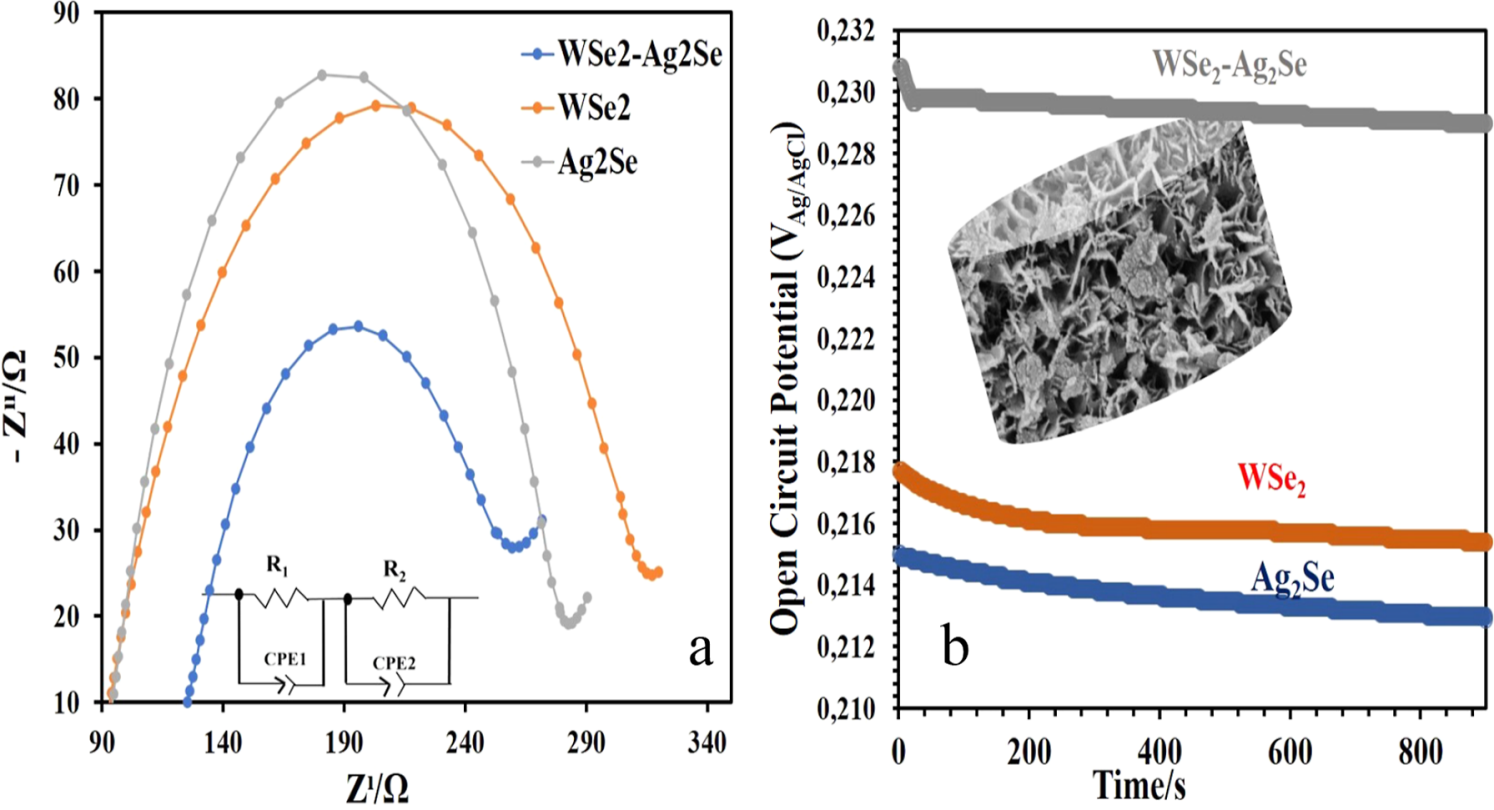
(a) Comparative Nyquist data of WSe_2_, AgSe_2_, and WSe_2_–AgSe_2_ hybrid nanostructures at 10 mM K_3_Fe­(CN)_6_, 10 mM K_4_Fe­(CN)_6_, and 0.1 M KCl. Frequency range: 0.1–105 Hz. (b) open-circuit potential (OCP) curves, E­(OCP)/time plots for WSe_2_, AgSe_2_, and WSe_2_–AgSe_2_ samples at 10 mM K_3_Fe­(CN)_6_, 10 mM K_4_Fe­(CN)_6_, and 0.1 M KCl.

Open circuit potential (OCP) curves are shown in Figure 7b, which show the relationship between electrode potential and immersion time (900 s) in electrolyte solution at 10 mM K_3_Fe­(CN)_6_, 10 mM K_4_Fe­(CN)_6_ and 0.1 M KCl. The OCP values (*E*_ocp_) for both samples gradually decrease to reach a steady state. Initially, the OCP decreased slowly and gradually reached a constant value for all tested samples. This clearly shows that the formation of the oxide layer starts within a few seconds and becomes constant over time. OCP measurements exhibited a more positive and stable potential for WSe_2_/Ag_2_Se (*E*_ocp_ ≈ 0.232 → 0.230 V) compared with WSe_2_ (0.218 → 0.217 V) and Ag_2_Se (0.215 → 0.214 V), indicating improved electrochemical stability and a stable surface passivation layer. Evaluation of the obtained electron transfer values revealed that the electron transfer resistance of the WSe_2_–Ag_2_Se thin film was relatively low, attributed to the increased active surface area and the enhanced electrochemical activity. Therefore, the impedance analysis further corroborates the H_2_-current response results. The initial decrease in Eocp is associated with the formation of a surface passive layer. For all three samples, Eocp briefly decreases during the first 100 s and subsequently stabilizes at a relatively constant value, indicating the formation of a protective passive layer. Accordingly, the higher potential difference observed in WSe_2_–Ag_2_Se clearly demonstrates that its passivation capability is superior to that of the other electrodes tested in this study.^48–51^

The hydrogen sensing mechanism of the WSe_2_/Ag_2_Se nanostructured electrodes can be explained in terms of surface adsorption, redox reactions, and interfacial charge transfer at the semiconductor–gas interface. In ambient air, oxygen molecules are chemisorbed onto the electrode surface and capture electrons from the conduction band to form negatively charged oxygen species (O_2_^–^, O^–^, or O^2–^), as described by the following reactions

1 $$O_{2}\left(g\right)+e^{-}\to O_{2}^{-}\;\left(ads\right)$$

2 $$O_{2}^{-}\;\left(ads\right)+e^{-}\to 2O^{-}\;\left(ads\right)$$

3 $$O^{-}\;\left(ads\right)+e^{-}\to O^{2-}\;\left(ads\right)$$

These oxygen ions withdraw electrons from the conduction band, generating a surface depletion layer and decreasing the baseline current. Upon exposure to H_2_, hydrogen molecules are dissociatively adsorbed onto the catalytic Ag_2_Se/WSe_2_ surface and react with the adsorbed oxygen ions to form water, simultaneously releasing electrons back into the conduction band, as shown in eqs 4 and 5)­

4 $$H_{2}\;\left(g\right)\to 2H\;\left(ads\right)$$

5 $$2H\;\left(ads\right)+O^{-}\;\left(ads\right)\to H_{2}O\;\left(g\right)+e^{-}$$

This reaction decreases the surface depletion width and enhances the conductivity, resulting in a measurable increase in the current signal. As shown in Figure 7a, the current density increased from 1.03 mA cm^–2^ for WSe_2_ and 5.68 mA cm^–2^ for Ag_2_Se to 7.89 mA cm^–2^ for the WSe_2_/Ag_2_Se hybrid electrode at 5000 ppm of H_2_ and 25 °C. The response exhibited a clear concentration dependence (Figure 7b). The current signal rose from 3.12 mA cm^–2^ at 1000 ppm to 5.46 mA cm^–2^ at 2500 ppm, and 7.89 mA cm^–2^ at 5000 ppm, indicating efficient gas–surface interaction and a strong catalytic effect. Temperature-dependent tests (Figure 7c) further confirmed that the current response at 150 °C (8.21 mA cm^–2^) was only slightly higher than that at 25 °C (7.89 mA cm^–2^), suggesting that the hybrid electrode exhibits efficient hydrogen detection even at room temperature with minimal thermal activation. This finding demonstrates the electrode’s high intrinsic conductivity and rapid charge transfer capability. In summary, hydrogen adsorption and the associated redox reactions (eqs 1–5) govern the sensing response of the WSe_2_/Ag_2_Se electrode.

The heterojunction formed between WSe_2_ and Ag_2_Se promotes efficient interfacial charge transfer, leading to a rapid, reversible, and stable sensing response. This synergistic effect accounts for the enhanced current response, lower charge transfer resistance, and excellent performance even at low operating temperatures. As summarized in Table 1, the WSe_2_/Ag_2_Se hybrid sensor combines low-temperature operation, high current response, and fast reversible sensing, outperforming previously reported hydrogen sensors due to strong interfacial interactions that enhance charge transport and electron transfer. The comparative analysis reveals that most previously reported hydrogen sensors typically operate at elevated temperatures (≥200 °C) to attain satisfactory sensitivity and response characteristics. However, such high-temperature operation inevitably increases power consumption and accelerates material degradation, thereby compromising long-term stability and limiting practical integration into portable or flexible electronic systems.

**Table 1:** Comparison of Existing Hydrogen Sensors and the Present WSe_2_/Ag_2_Se-Based Sensor

sensor material	synthesis/preparation method	operating temperature	H_2_ concentration range (ppm)	response (current or resistance change)	response/recovery time (s)	references
Pd–WO_3_ thin film	sputtering	200 °C	100–1000	Δ*R* ∼ 2.5 × 103 Ω	50/45	^49^
MoS_2_/WSe_2_ nanosheets	mechanical	RT	50–500	Δ*I* ∼ 0.8 μA	60/70	^24^
WS_2_ 2D nanosheets	chemical vapor deposition (CVD)	RT	100–500	Δ*I* ∼ 1.2 μA	45/50	^25^
metal oxides	various	150–300 °C	50–5000	variable	30–120	^22,23^
WSe_2_/Ag_2_Se hybrid (present work)	electrochemical deposition	25–150 °C	1000–5000	7.89 mA cm^–2^	35/40	this work

In contrast, the electrochemically synthesized WSe_2_/Ag_2_Se hybrid electrode demonstrates efficient hydrogen sensing performance at significantly lower operating temperatures (25–150 °C), while retaining rapid, reversible, and stable response behavior. The hybrid sensor achieves a high current response of 7.89 mA cm^–2^ at 5000 ppm of H_2_, indicating enhanced charge carrier mobility and strong interfacial charge transfer between the WSe_2_ and Ag_2_Se phases. These synergistic effects underscore the promise of the WSe_2_/Ag_2_Se hybrid as a low-power, cost-effective, and durable hydrogen sensing platform, well-suited for next-generation, real-time, and energy-efficient sensing applications.

CV data of WSe_2_, Ag_2_Se, and WSe_2_–Ag_2_Se electrodes electrochemically coated on ITO electrodes, taken at different scan rates (from 10 mV/s to 70 mV/s) for each of them in 0.1 M KCl electrolyte containing 10 mM Fe­(CN)_6_^3–^/Fe­(CN)_6_^4–^ at room temperature are shown in Figure 8. The cyclic voltammetry (CV) curves of the WSe_2_, Ag_2_Se, and WSe_2_/Ag_2_Se electrodes, recorded at scan rates between 5 and 150 mV s^–1^, exhibit well-defined redox peaks, confirming the electrochemical activity of each material.

**Figure 8:**
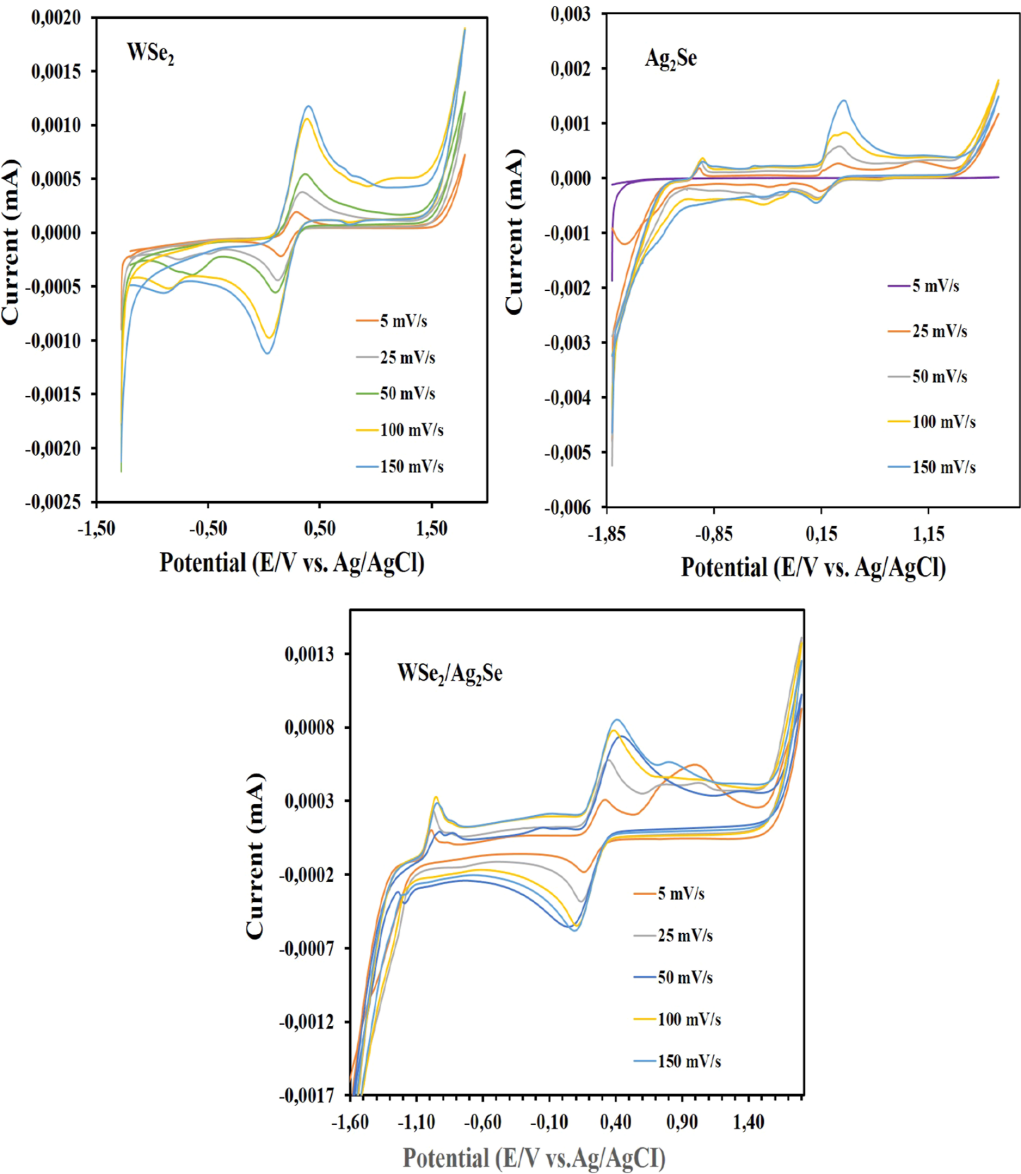
Cyclic voltammetry (CV) measurements of WSe_2_, Ag_2_Se, and WSe_2_–Ag_2_Se electrodes recorded at scan rates ranging from 5 to 150 mV·s^–1^.

The anodic and cathodic peak currents increase linearly with scan rate, indicating a surface-controlled redox process. For the pure WSe_2_ electrode, the anodic peak appeared around +1.35 V with a current of approximately 1.8 μA, while for Ag_2_Se it was located near +1.10 V with a current of about 2.4 μA. In contrast, the WSe_2_/Ag_2_Se hybrid electrode showed a more intense anodic peak at approximately +1.25 V, with a significantly higher current density of around 3.8 μA.

The hybrid electrode exhibits a markedly higher redox current density and improved electrochemical reversibility compared to the individual WSe_2_ and Ag_2_Se electrodes. This enhancement is attributed to the synergistic interfacial interaction between WSe_2_ nanosheets and Ag_2_Se nanoparticles, which facilitates rapid electron transport and increases the number of active surface sites. The well-overlapped redox peaks confirm the structural and electrochemical stability of the hybrid system. Consequently, the WSe_2_/Ag_2_Se hybrid electrode demonstrates superior electrocatalytic behavior toward H_2_ adsorption and dissociation, leading to enhanced hydrogen gas sensitivity and excellent potential for application in low-temperature H_2_ sensing devices.

## 4 Conclusions

In conclusion, it revealed that the size of WSe_2_–Ag_2_Se nanostructures can be tuned by controlling the deposition time. The electrochemical coupling of WSe_2_ with a selected conductive material (Ag_2_Se) significantly enhanced the conductivity and accelerated the overall catalytic reactivity. The catalytic performance and conductivity property were greatly enhanced by growing stable WSe_2_ nanostructures by electrodeposition method through a stabilized interface contact with Ag_2_Se nanostructured electrodes, improving the active electrocatalyst’s electronic structure, property, and performance. Meanwhile, the surface area of WSe_2_–Ag_2_Se electrodes is also increased, and abundant active sites are exposed by the reduction of layer density along with the formation of interface active spots. WSe_2_–Ag_2_Se nanostructured electrodes activate electrochemical processes, while their conductivity related to the synergistic effect resulting from the interaction of both components improves the overall catalytic performance of the systems. By taking advantage of its conductive structure and ionic mobility, it can be readily used to improve electroactive materials with poor conductivity for energy generation and storage purposes, reducing the layer density and increasing the surface area. WSe_2_–Ag_2_Se hybrid nanostructures are seen as promising for enhancing performance.
